# Supplementary material for: A new species of Brachycephalus (Anura: Brachycephalidae) from southern Brazil
Source: PeerJ. 2018 Oct 3;6:e5683. doi: 10.7717/peerj.5683 (PMC6174073; doi:10.7717/peerj.5683)
Supplement: Supplemental Information 2 [file peerj-06-5683-s002.docx]

Table S1. Accession numbers of the sequences used in the present study.

| Species | Accession number |
| --- | --- |
| *Brachycephalus actaeus* | MG889448 |
| *Brachycephalus albolineatus* | MG889434 |
| *Brachycephalus auroguttatus* | KX025369 |
| *Brachycephalus boticario* | KX025377 |
| *Brachycephalus brunneus* | HQ435691 |
| *Brachycephalus curupira* | KX025382 |
| *Brachycephalus didactylus* | HQ435692 |
| *Brachycephalus ferruginus* | HQ435695 |
| *Brachycephalus fuscolineatus* | KX025343 |
| *Brachycephalus izecksohni* | HQ435696 |
| *Brachycephalus leopardus* | KX025247 |
| *Brachycephalus mariaeterezae* | KX025353 |
| *Brachycephalus mirissimus* | MH136570 |
| *Brachycephalus olivaceus* | KX025325 |
| *Brachycephalus pernix* | HQ435698 |
| *Brachycephalus pombali* | HQ435700 |
| *Brachycephalus quiririensis* | KX025322 |
| *Brachycephalus tridactylus* | KX025391 |
