# Supplementary material for: A new species of Brachycephalus (Anura: Brachycephalidae) from southern Brazil
Source: PeerJ. 2018 Oct 3;6:e5683. doi: 10.7717/peerj.5683 (PMC6174073; doi:10.7717/peerj.5683)
Supplement: Supplemental Information 3 [file peerj-06-5683-s003.docx]

Table S2. Comparison of the features used to describe the advertisement call of *Brachycephalus*.

| Feature | *B. pernix* group | | | | | *B. ephippium* group | | | | | | | | *B. didactylus* group | | |
| --- | --- | --- | --- | --- | --- | --- | --- | --- | --- | --- | --- | --- | --- | --- | --- | --- |
|  | *B. mirissimus* | *B. actaeus* | *B. albolineatus* | *B. tridactylus* | *B. crispus* | | *B. darkside* | *B. ephippium* | *B. ephippium* | *B. pitanga* | *B. pitanga* | *B. pitanga* | *B. hermogenesi* | | *B. sulfuratus* |  |
| Call duration (s) | 111.834 ± 46.604 (37.700–255.205) [25/11] | 0.04 ± 0.01 (0.03–0.04) [110/6]^1^ | 88.367 ± 35.733 (39.933–191.141) [24/16] | 0.11 ± 0.02 (?–? ) [?/17]^1^ | ? ± ? (?–300) [5/?] | | 30.4 ± 25.3 (2.9–66.2) [7/5] | ? ± ? (120–360) [?/?] |  |  |  |  | ? ± ? (0.2–1.9) [?/?]^2^ | | 1.8 ± 0.2 (1.5–2.3) [95/11] |  |
| Call rate (calls per second) |  | 0.20 ± 0.07 (0.13–0.30) [110/6] |  |  |  | |  |  |  |  |  |  | 0.19 ±? (?–?) [?/?]^2^ | |  |  |
| Interval between calls (s) |  | 5.42 ± 1.83 (3.27–7.68) [104/6] |  |  |  | | 6.2, 11.2 [2/?] |  |  |  |  |  |  | | 5.1 ± 1.4 (3.1–7.4) [95/11] |  |
| Note rate (notes per minute) | 11.694 ± 2.119 (7.484–15.935) [30/11] |  | 11.439 ± 3.216 (5.891–18.088) [24/16] |  |  | | 211.4 ± 25.6 (186.4–243.4) [5/?] |  |  | 159 ± 11 (?–?) [?/2] |  |  |  | |  |  |
| Note rate (notes per second) |  |  |  | 0.16 ± 0.03 (?–?) [11/?] | 1.67 ± 0.09 (?–?) [5/?] | |  |  |  |  |  |  | 1.09 ± ? (?–?) [?/?]^2^ | | 0.2 ± 0.0 (0.1–0.3) [485/11] |  |
| Pulse rate (pulses per second) |  | 0.42 ± 0.15 (0.26–0.63) [229/6] |  |  | 17.4 ± 2.12 (?–?) [5/?] | | 56.9 ± 4.9 (36.8–78.4) [790/5] |  |  | 62 ± 8 (?–?) [?/2]^3^ |  |  |  | | 9.3 ± 1.8 (6.1–12.3) [?/11] |  |
| Number of notes per call | 23.552 ± 10.287 (6–52) [29/12] |  | 17.26 ± 6.38 (8–29) [27/16] | 1 |  | | 114 ± 97.1 (9–253) [7/5] |  |  |  |  |  | ? ± ? (1–7) [?/?]^2^ | | 5.3 ± 0.9 (4–7) [485/11] |  |
| Number of pulses per notes |  | ? ± ? (2–3) [?/?] |  |  |  | |  |  |  |  |  |  |  | |  |  |
| Number of pulses per isolated notes | 1.691 ± 0.475 (1–3) [362/12] |  | 2.00 ± 0.595 (1–3) [323/20] | 0 | 10 ± 1.19 (7–12) [100/5] | | 6.3 ± 0.7 (5–8) [790/5] | 12 ± 1.96 (5–15) [57/?] |  | 11.1 ± 1.2 (?–?) [?/2] | 10.86 ± 1.62 (6.90–14.30) [?/?] |  |  | | 8.8 ± 1.3 (7–11) [?/11] |  |
| Number of pulses per note in note groups | 1.958 ± 0.325 (1–3) [308/10] |  | 2.70 ± 0.459 (2–3) [230/16] |  |  | |  |  |  |  |  |  |  | |  |  |
| Number of pulses in each note groups | 3.916 ± 0.604 (2–6) [154/10] |  | 5.40 ± 0.825 (4–6) [115/16] |  |  | |  |  |  |  |  |  |  | |  |  |
| Note duration of isolated notes (s) | 0.010 ± 0.007 (0.002–0.027) [115/10] |  | 0.020 ± 0.007 (0.002–0.037) [96/19] | 0.11 ± 0.02 (?–?) [?/17] | 0.28 ± 0.02 (?–?) [100/5] | | 0.111 ± 0.014 (0.083–0.163) [790/5] | 0.112 ± 0.006 (0.093–0.125) [19/?] |  | 0.170 ± 0.013 (?–?) [?/2] | 0.19 ± 0.03 (0.15–0.25) [400/40] |  |  | | 0.195 ± 0.013 (0.131–0.233) [485/11] |  |
| Duration of note groups (s) | 0.426 ± 0.044 (0.361–0.590) [56/10] |  | 0.465 ± 0.053 (0.360–0.578) [62/16] |  |  | |  |  |  |  |  |  |  | |  |  |
| Pulse duration (s) |  |  |  |  | 0.027 ± 0.004 (?–?) [517/5] | |  |  |  |  |  |  |  | | 0.024 ± 0.005 (0.02–0.03) [?/11] |  |
| Inter-note interval in isolated notes (s) | 5.815 ± 1.328 (3.919–10.625) [84/10] |  | 6.663 ± 1.705 (4.092–12.248) [62/15] |  | 0.35 ± 0.02 (?–?) [100/5] | | 0.159 ± 0.014 (0.122–0.215) [783/5] | 0.134 ± 0.007 (0.123–0.149) [18/?] |  |  | 0.28 ± 0.05 (0,20–0.43) [400/40] |  |  | |  |  |
| Inter-note group interval (s) | 7.022 ± 1.133 (5.321–10.930) [34/10] |  | 6.871 ± 1.768 (4.322–10.678) [32/13] |  |  | |  |  |  |  |  |  |  | |  |  |
| Inter-note interval within note groups (s) | 0.389 ± 0.030 (0.346–0.490) [56/10] |  | 0.412 ± 0.050 (0.319–0.526) [55/16] |  |  | |  |  |  |  |  |  |  | |  |  |
| Note dominant frequency (kHz) | 6.645 ± 0.272 (6.000–7.230) [227/10 | 6.9 ± 0.3 (6.6–7.3) [110/6] | 6.376 ± 0.304 (5.340–7.321) [256/10] | 4.8 ± 0.2 (?–?) [?/17] | 4.6 ± 0.19 (?–?) [100/5] | | 3.382 ± 0.185 (2.856–3.797) [790/5]^4^ |  | 3.94 ± 0.24 (?–? ) [?/5] | 4.9 ± 0.2 (?–?) [?/2] | 4.816 ± 0.414 (4.311–5.550) [400/40] | 5.43 ± 0.30 (?–?) [?/8] |  | | 6.7 ± 0.3 (6.2–7.2) [?/11] |  |
| Call dominant frequency (kHz) |  |  |  |  |  | |  |  |  |  |  |  | ?–? (6.8 ± 0.8) [5/?]^5^ | |  |  |
| Highest frequency (kHz) | 8.311 ± 0.518 (7.143–10.060) [227/10] | 9.1 ± 0.4 (8.5–9.7) [110/6] | 8.437 ± 0.492 (7.113–9.852) [145/19] | ? ± ? (6.4) [?/17]^6^ | 5.7 ± 0.17 (?–?) [100/5]^6^ | |  | ? ± ? (5.3) [?/?]^6^ |  |  |  |  |  | | 9.3 ± 0.3 (8.2–10.3) [?/11]^6^ |  |
| Lowest frequency (kHz) | 4.369 ± 0.767 (2.667–5.841) [277/10] | 4.8 ± 0.7 (3.9–5.9) [110/6] | 4.066 ± 0.448 (3.092–5.212) [145/19] | ? ± ? (3.2) [?/17]^6^ | 3.5 ± 0.19 (?–?) [100/5]^6^ | |  | 3.4 (? ± ?) [?/?]^6^ |  |  |  |  |  | | 4.9 ± 0.3 (4.5–5.5) [?/11]^6^ |  |
| 5%–95% frequency^7^ |  |  |  |  |  | | ? ± ? (2.484–5.766) [?/?] |  |  |  |  |  |  | |  |  |
| “Highest sound pressure” (dB) |  |  |  | 110 ± 5.6 (?–?) [?/17] |  | |  |  | 47.0 ± 5.7 (?–?) [3/?] | ? ± ? (56–66) [4/?] |  | 57.6 ± 1.8 (?–?) [8/?] |  | |  |  |
| Approach (*sensu* Köhler *et al.* 2017) | note-centered | call-centered | note-centered | call-centered | note-centered | | note-centered | note-centered | not applicable | note-centered^2^ | note-centered | not applicable | note-centered^2^ | | note-centered |  |
| Source | this study | Monteiro *et al.* (2018) | Bornschein *et al*. (2018) | Garey *et al.* (2012) | Condez *et al.* (2014) | | Guimarães *et al.* (2017) | Pombal Jr., Sazima & Haddad (1994) | Goutte *et al.* (2017) | Araújo *et al.* (2012) | Tandel *et al.* (2014) | Goutte *et al.* (2017) | Verdade *et al.* (2008) | | Condez *et al.* (2016) |  |

**Notes:**

Values are expressed by: mean ± SD (range) [sample/specimens]. Abbreviation: SD = standard deviation.

^1^Represents note duration under note-centered approach.

^2^Note-centered approach and call-centered approach probably mixed in this measurement.

^3^The unit of measure was erroneously cited as Hz.

^4^Feature cited as “peak frequency” by Guimarães *et al.* (2017) but refers to our dominant frequency.

^5^We are not sure if in the measurement was not mixed with note dominant frequency.

^6^The measurement procedure has not been explained and data may be not comparable.

^7^Feature cited as “dominant frequency” by Guimarães *et al.* (2017).

References

Araújo, C.B. de, Guerra, T.J., Amatuzzi, C.O.M. & Campos, L.A. (2012) Advertisement and territorial calls of *Brachycephalus pitanga* (Anura: Brachycephalidae). *Zootaxa*, 3302, 66–67.

Bornschein, M.R., Ribeiro, L.F., Rollo Jr., M.M., Confetti, A.E. & Pie, M.R. (2018) Advertisement call of *Brachycephalus albolineatus* (Anura: Brachycephalidae). *PeerJ*, 6, e5273.

Condez, T.H., Clemente-Carvalho, R.B.G., Haddad, C.F.B. & Reis, S.F. dos (2014) A new species of *Brachycephalus* (Anura: Brachycephalidae) from the highlands of the Atlantic Forest, Southeastern Brazil. *Herpetologica*, 70, 89–99.

Condez, T.H., Monteiro, J.P. de C., Comitti, E.J., Garcia, P.C. de A., Amaral, I.B. & Haddad, C.F.B. (2016) A new species of flea-toad (Anura: Brachycephalidae) from southern Atlantic Forest, Brazil. *Zootaxa*, 4083, 40–56.

Garey, M.V., Lima, A.M.X., Hartmann, M.T. & Haddad, C.F.B. (2012) A new species of miniaturized toadlet, genus *Brachycephalus* (Anura: Brachycephalidae), from Southern Brazil. *Herpetologica*, 68, 266–271.

Goutte, S., Mason, M.J., Christensen-Dalsgaard, J., Montealegre-Z, F., Chivers, B.D., Sarria-S, F.A., Antoniazzi, M.M., Jared, C., Sato, L.A. & Toledo, L.F. (2017) Evidence of auditory insensitivity to vocalization frequencies in two frogs. *Scientific Reports*, 7, 12121.

Guimarães, C.S., Luz, S., Rocha, P.C. & Feio, R.N. (2017) The dark side of pumpkin toadlet: a new species of *Brachycephalus* (Anura: Brachycephalidae) from Serra do Brigadeiro, southeastern Brazil. *Zootaxa*, 4258, 327–344.

Monteiro, J.P. de C., Condez, T.H., Garcia, P.C. de A., Comitti, E.J., Amaral, I.B. & Haddad, C.F.B. (2018) A new species of *Brachycephalus* (Anura, Brachycephalidae) from the coast of Santa Catarina State, southern Atlantic Forest, Brazil. *Zootaxa*, 4407(4), 483–505.

Pombal Jr., J.P., Sazima, I. & Haddad, C.F.B. (1994) Breeding behavior of the pumpkin toadlet, *Brachycephalus ephippium* (Brachycephalidae). *Journal of Herpetology*, 28, 516–519.

Tandel, M. da C.F.F., Loibel, S., Oliveira, E.G. de & Haddad, C.F.B. (2014) Diferenciação de 3 tipos de vocalizações (cantos) na espécie *Brachycephalus pitanga*. *Revista da Estatística da Universidade Federal de Ouro Preto*, 3, 374–386.

Verdade, V.K., Rodrigues, M.T., Cassimiro, J., Pavan, D., Liou, N. & Lange, M. (2008) Advertisement call, vocal activity, and geographic distribution of *Brachycephalus hermogenesi* (Giaretta and Sawaya, 1998) (Anura, Brachycephalidae). *Journal of Herpetology*, 42, 542–549.
